# Supplementary material for: Notch and TLR signaling coordinate monocyte cell fate and inflammation
Source: eLife. 2020 Jul 29;9:e57007. doi: 10.7554/eLife.57007 (PMC7413669; doi:10.7554/eLife.57007)
Supplement: Supplementary file 1. — Lin: CD3, CD45R/B220, CD19, NK1.1, Ly6G, Ter119. [file elife-57007-supp1.doc]

| **Population** | **Phenotype** |
| --- | --- |
| Ly6C^hi^ | CD45^+^Lin^-^CD117^-^CD11b^+^CX_3_CR1^+^Ly6C^hi^F4/80^lo/-^CD11c^-^MHC-II^lo/-^CD43^-^ |
| Ly6C^lo^ | CD45^+^Lin^-^CD117^-^CD11b^+^CX_3_CR1^+^Ly6C^lo/-^F4/80^lo/-^CD11c^lo^MHC-II^lo/-^CD43^+^ |
| MHC-II^+^ cells (Ly6C^lo^) | CD45^+^Lin^-^CD117^-^CD11b^+^CX_3_CR1^+^Ly6C^lo/-^F4/80^lo/-^CD11c^-^MHC-II^+^CD43^-^ |
| MF | CD45^+^Lin^-^CD117^-^CD11b^+^CX_3_CR1^+^Ly6C^lo/-^F4/80^hi^CD115^+^ |
| DC | CD45^+^Lin^-^CD117^-^CD11b^+^CX_3_CR1^+^Ly6C^lo/-^F4/80^lo/-^CD11c^+^MHC-II^+^CD43^-^ |
| GC | CD45^+^Lin^+^CD11b^+^CX_3_CR1^-^Ly6C^lo^F4/80^-^CD11c^-^MHC-II^-^ |
