# Supplementary material for: Notch and TLR signaling coordinate monocyte cell fate and inflammation
Source: eLife. 2020 Jul 29;9:e57007. doi: 10.7554/eLife.57007 (PMC7413669; doi:10.7554/eLife.57007)
Supplement: Supplementary file 2. [file elife-57007-supp2.doc]

| Diseases or Functions Annotation | *P*-value | B-H *P*-value | Activation z-score | Bias-corrected z-score | # Molecules |
| --- | --- | --- | --- | --- | --- |
| Systemic autoimmune syndrome | 4,36E-17 | 1,11E-13 | 1,214 | 1,176 | 40 |
| Insulin-dependent  diabetes mellitus | 7,71E-15 | 4,93E-12 | 0,820 | 0,679 | 32 |
| Rheumatoid arthritis | 8,53E-10 | 1,05E-07 |  |  | 15 |
| Experimental autoimmune encephalomyelitis | 3,60E-08 | 2,83E-06 | 2,244 | 2,009 | 26 |
| Abnormal morphology of immune system | 1,43E-07 | 8,70E-06 |  |  | 24 |
