# Supplementary material for: Notch and TLR signaling coordinate monocyte cell fate and inflammation
Source: eLife. 2020 Jul 29;9:e57007. doi: 10.7554/eLife.57007 (PMC7413669; doi:10.7554/eLife.57007)
Supplement: Supplementary file 3. [file elife-57007-supp3.doc]

| **GO term of biological process from MSigDB** | **SIZE** | **ES** | **NES** | **NOM *P*-val** | **FDR** |
| --- | --- | --- | --- | --- | --- |
| GO VESICLE MEDIATED TRANSPORT | 38 | 0.582 | 2.148 | 0.00000 | 0.00206 |
| GO DEFENSE RESPONSE | 43 | 0.568 | 2.115 | 0.00000 | 0.00209 |
| GO INFLAMMATORY RESPONSE | 26 | 0.587 | 2.008 | 0.00000 | 0.01516 |
| GO RESPONSE TO BACTERIUM | 20 | 0.613 | 2.002 | 0.00000 | 0.01242 |
| GO ENDOCYTOSIS | 23 | 0.579 | 1.961 | 0.00111 | 0.01822 |
| GO RESPONSE TO MOLECULE OF BACTERIAL  ORIGIN | 17 | 0.618 | 1.961 | 0.00228 | 0.01518 |
| GO ION HOMEOSTASIS | 16 | 0.636 | 1.958 | 0.00234 | 0.01316 |
| GO CHEMICAL HOMEOSTASIS | 22 | 0.575 | 1.943 | 0.00000 | 0.01321 |
| GO CELLULAR RESPONSE TO CYTOKINE  STIMULUS | 18 | 0.616 | 1.929 | 0.00114 | 0.01540 |
| GO RESPONSE TO LIPID | 23 | 0.551 | 1.857 | 0.00554 | 0.03973 |
| GO RESPONSE TO CYTOKINE | 22 | 0.558 | 1.857 | 0.00112 | 0.03621 |
| GO LEUKOCYTE MIGRATION | 16 | 0.602 | 1.852 | 0.00471 | 0.03536 |
| GO SECRETION BY CELL | 15 | 0.608 | 1.846 | 0.00240 | 0.03533 |
| GO RESPONSE TO BIOTIC STIMULUS | 25 | 0.538 | 1.828 | 0.00216 | 0.04046 |
| GO REGULATION OF PROTEOLYSIS | 17 | 0.580 | 1.818 | 0.00349 | 0.04204 |
| GO REGULATION OF HYDROLASE ACTIVITY | 35 | 0.486 | 1.803 | 0.00106 | 0.04568 |
| GO CELLULAR CHEMICAL HOMEOSTASIS | 18 | 0.567 | 1.784 | 0.00335 | 0.05165 |
| GO REGULATION OF CYTOKINE PRODUCTION | 22 | 0.536 | 1.782 | 0.00444 | 0.04965 |
| GO POSITIVE REGULATION OF HYDROLASE  ACTIVITY | 27 | 0.502 | 1.770 | 0.00532 | 0.05330 |
