# Supplementary material for: Notch and TLR signaling coordinate monocyte cell fate and inflammation
Source: eLife. 2020 Jul 29;9:e57007. doi: 10.7554/eLife.57007 (PMC7413669; doi:10.7554/eLife.57007)
Supplement: Supplementary file 6. [file elife-57007-supp6.doc]

| **Antibody/Dye** | **Clone** | **Company** |
| --- | --- | --- |
| CD3 | 17A2 | BioLegend |
| Ter119 | Ter119 | BioLegend |
| CD45R/B220 | RA3-6B2 | BioLegend |
| Ly6G | 1A8 | BioLegend |
| CD19 | 6D5 | BioLegend |
| NK1.1 | PK136 | BioLegend |
| CD117 | 2B8 | BioLegend |
| CD115 | AFS98 | BioLegend |
| CD11b | M1/70 | BioLegend |
| Ly6C | HK1.4 | BioLegend |
| F4/80 | BM8 | BioLegend |
| CD11c | N418 | BioLegend |
| I-A/I-E | M5/114.15.2 | BioLegend |
| CD64 | X54-5/7.1 | BioLegend |
| CD45 | 30-F11 | BioLegend |
| CD45.1 | A20 | BioLegend |
| CD45.2 | 104 | BioLegend |
| CX_3_CR1 | SA011F11 | BioLegend |
| MerTK | DS5MMER | eBioscience |
| CD43 | S7 | BD Pharmingen |
| Streptavidin-PE-Dazzled594 |  | BioLegend |
| AnnexinV-APC |  | BioLegend |
| 7AAD |  | BioLegend |
| Propidium Iodide |  | Sigma |
